# Spindle Cell Renal Neoplasms: A Pathological Case Report on Primary Renal Leiomyosarcoma and Sarcomatoid Renal Cell Carcinoma

**DOI:** 10.7759/cureus.46449

**Published:** 2023-10-03

**Authors:** Hristo Popov, Lilyana Petkova, George S Stoyanov

**Affiliations:** 1 General and Clinical Pathology, Forensic Medicine and Deontology, Medical University of Varna, Varna, BGR; 2 Pathology, Complex Oncology Center, Shumen, BGR

**Keywords:** leiomyosarcoma, sarcomatoid tumors, renal cell carcinoma, oncology, kidney

## Abstract

Renal oncopathology in adults, as a field of pathology, is dominated by a single entity - clear cell renal cell carcinoma (RCC) with other entries, such as urothelial carcinoma of the renal pelvis, angiosarcoma, and others being extremely rare. Herein, we report two histopathological cases with differential diagnoses of spindle cell renal neoplasms. The first patient, a 42-year-old male, presented with new-onset right-sided abdominal flank pain, and imaging showed a 12 cm renal tumor. Histopathology showed a spindle cell neoplasm, with significant mitotic activity and giant cell, with immunohistochemistry being positive for caldesmon and vimentin, focally for smooth muscle actin (SMA). No reaction was noted for pan-cytokeratin (CK AE1/AE3), epithelial membrane antigen (EMA), cytokeratin (CK) 7, cluster of differentiation (CD) 117, soluble 100 protein (S100), human melanoma black (HMB) 45, Melan A, CD10, and desmin. Due to peculiar histomorphology and the immunophenotype, the tumor was interpreted as primary renal leiomyosarcoma. Due to continuous outpatient consultations, treatment initiation was delayed, and three months later, the patient had already developed an 87 mm local recurrence and liver metastasis. The second patient, a 53-year-old male, presented to our institution for consultation of an already excised renal tumor, diagnosed as an incidental finding on a prophylactic abdominal ultrasound. The tumor presented for consultation histologically grew as intertwining bundles of spindle cells with polymorphic hyperchromic nuclei with prominent nucleoli and had extensive areas with necrosis. Immunohistochemically, the tumor diffusely expressed CK AE1/AE3 and caldesmon and had a patchy reaction for EMA and CD10. The SMA, desmin, CD117, and CK7 reactions were negative; hence, the tumor was interpreted as a spindle cell variety (sub-type) of clear RCC.

## Introduction

Spindle cell tumors of the kidney are rare and challenging histopathological entries and usually require a broad differential diagnosis [1,2]. Of this morphological group, the most common entries are the sarcomatoid (spindle cell) variant of renal cell carcinoma (RCC) and secondary renal involvement from primary retroperitoneal sarcomas, such as leiomyosarcoma, liposarcoma, angiosarcoma, and others [1].

Leiomyosarcoma of the kidney is a rare and aggressive mesenchymal tumor and accounts for about 1-2% of all malignant kidney tumors [3,4]. The average age at presentation is typically in the fourth or fifth decade of life, with tumor origin most widely attributed to smooth muscle cells of the renal blood vessels. Recurrence frequency is usually high, and the patient prognosis is dismal, with multiple distant metastases [3-7].

To take a correct approach to the treatment of these tumors, histological and immunohistochemical verification of the tumor mass is necessary, which can be performed with a core biopsy of the tumor preoperatively [8]. However, a definitive diagnosis is preferably made after gross excision of the tumor, as core biopsies may not represent the complete morphology of the tumor and may mislead the pathologist due to foci of aberrant immunohistochemical expression [2]. Furthermore, different nosological groups have not only different treatments and outcomes but also different grading and staging criteria [9-11].

Herein, we present two case reports of spindle cell renal neoplasms and a differential approach to the histopathological diagnosis.

## Case presentation

Case 1

The first case is of a 42-year-old male with a previously unremarkable medical history. He presented with new-onset, dull, right abdominal flank pain to his general practitioner. Laboratory tests were unremarkable, but with imaging modalities, ultrasound, and computer tomography (CT), a right-sided kidney tumor formation with the greatest size of 12 cm was detected, and the patient was referred for surgical treatment. An open nephrectomy was performed, with an unremarkable postoperative period.

The excised specimen grossly showed a renal tumor with diffuse infiltration and obliteration of the greater part of the renal parenchyma. The tumor formation on a section had a bacon-like appearance, grayish-white, and no infiltration was found in the renal hilus and perirenal adiposa.

**Figure 1:**
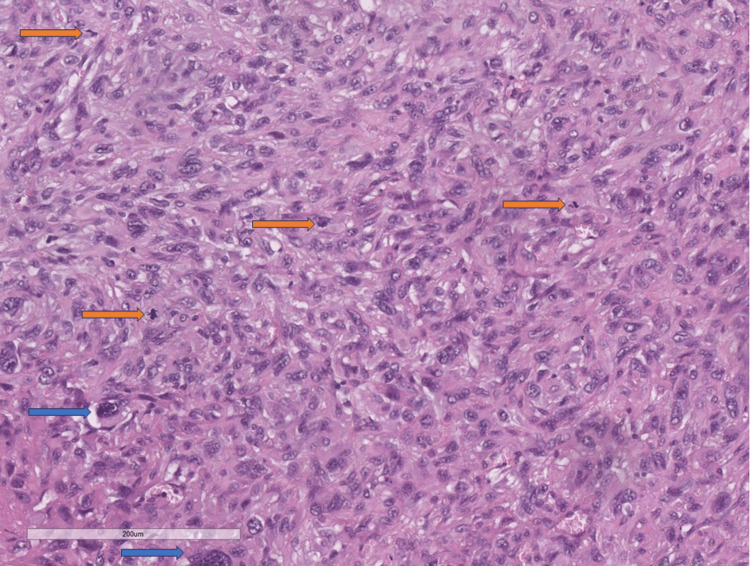
Histomorphology of the tumor, H&E stain, original magnification x200 Giant cells (blue arrows), mitotic figures (orange arrows) H&E: hematoxylin and eosin

**Figure 2:**
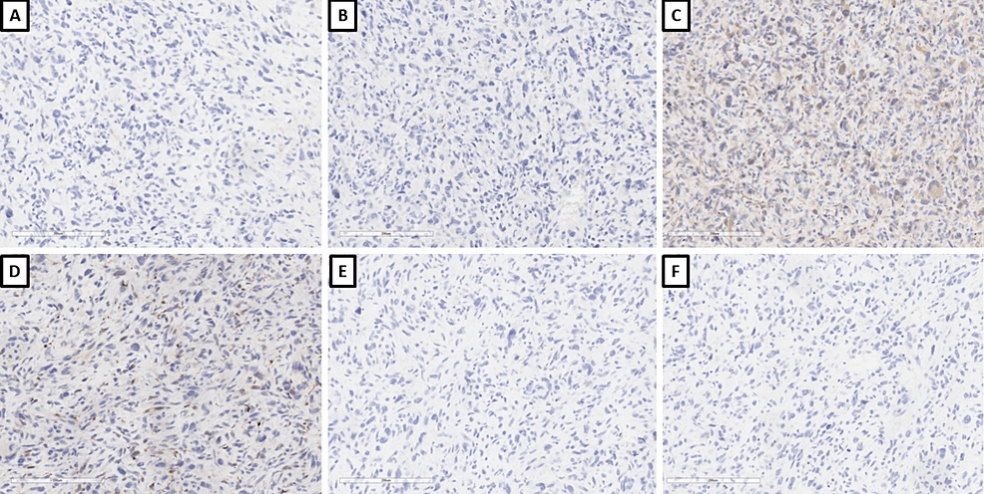
Immunophenotype, original magnifications x200 A: CD10; B: CK AE1/AE3; C: EMA; D: S100; E: HMB-45; F: PR CD: cluster of differentiation; CK AE1/AE3: pan-cytokeratin; EMA: epithelial membrane antigen; S100: soluble 100 protein; HMB-45: human melanoma black 45; PR: progesterone receptor

**Figure 3:**
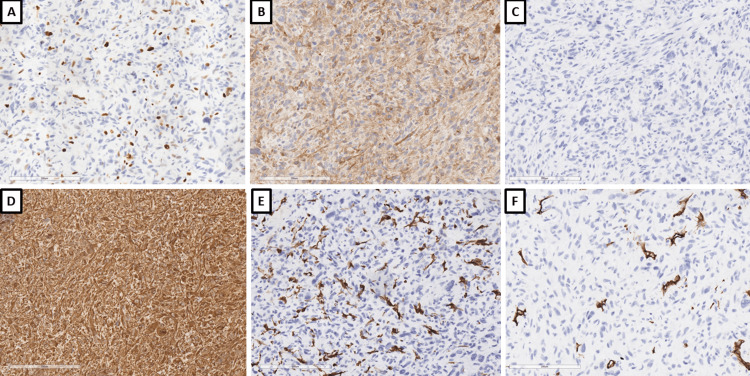
Immunophenotype, original magnifications x200 A: Ki-67; B: caldesmon; C: desmin; D: vimentin; E: SMA; F: CD34 Ki-67: proliferative index; SMA: smooth muscle actin; CD: cluster of differentiation

**Figure 4:**
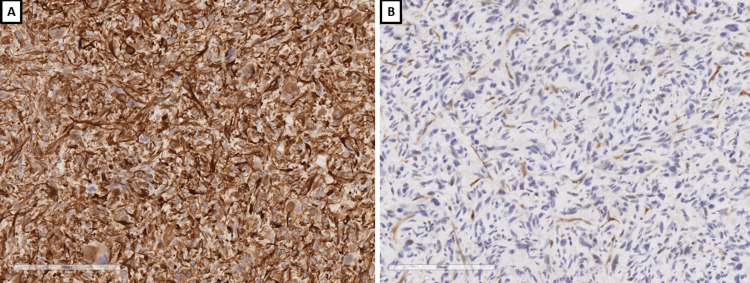
Immunophenotype, original magnifications x200 A: WT1; B: CD117 WT1: Wilms tumor 1; CD: cluster of differentiation

Histological examination showed a tumor formation with diffuse growth, represented by intertwining bundles of atypical cells with pronounced nuclear polymorphism, some multinucleated, forming storiform structures in places (Figure 1). Tumor cells showed a high mitotic index of over 20 mitoses per 10 x 400 high power fields (HPF) and areas of necrosis over 50% of the tumor tissue (Figure 1). Regarding differential diagnosis, sarcomatoid variants of renal cell carcinoma, primary leiomyosarcoma of the kidney, and liposarcoma developed on the basis of angiomyolipoma were suspected. The immunohistochemical panel applied included soluble 100 protein (S100), human melanoma black (HMB) 45, Melan A, pan-cytokeratin (CK AE1/AE3), epithelial membrane antigen (EMA), cluster of differentiation (CD) 10, smooth muscle actin (SMA), h-caldesmon, desmin, vimentin, cytokeratin (CK) 7, CD117, and Wilms tumor 1 (WT1) (Figures 2-4). The tumor cells were positive for h-caldesmon vimentin, focally for SMA, and a negative reaction by the tumor cells was reported for CK AE1/AE3, EMA, CK7, CD117, S100, HMB45, Melan A, CD10, and desmin (Figures 2-4). An aberrant cytoplasmic expression was noted for WT1 and EMA, which were regarded as undiagnostic (Figures 2-4). The tumor was interpreted as primary renal leiomyosarcoma and staged as per the eighth revision of the American Joint Committee on Cancer (AJCC) guidelines on soft tissue tumors - pT1 and graded (G) as G2 as per the Fédération Nationale des Centres de Lutte Contre le Cancer (FNCLCC) guidelines. Final staging with nodal and distant metastasis state was left to the oncologic committee.

Due to multiple outpatient consultations, treatment was postponed. The patient had a follow-up CT scan three months after surgery, with tumor progression noted. Subsequent positron emission tomography CT (PET-CT) showed a hypermetabolic lesion in the topographical area of the excised kidney measuring 87 mm at its greatest size, infiltrating the psoas major muscle and the inferior vena cava, as well as a similar lesion in the sixth liver segment measuring 26 mm. The patient was since lost to follow-up.

Case 2

The second case concerns a 53-year-old man who presented for consultation on an already excised renal tumor. Medical history included mild hypertension for 10 years under adequate medication control. Upon prophylactic abdominal ultrasound, the patient had an incidental finding of a renal tumor, and the finding was confirmed on CT. An open nephrectomy was performed, and a renal tumor with similar characteristics, as the one previously described, was noted, with visible invasion to the renal hilum noted. Histopathologically the tumor was interpreted as a spindle cell tumor and referred for immunohistochemistry.

**Figure 5:**
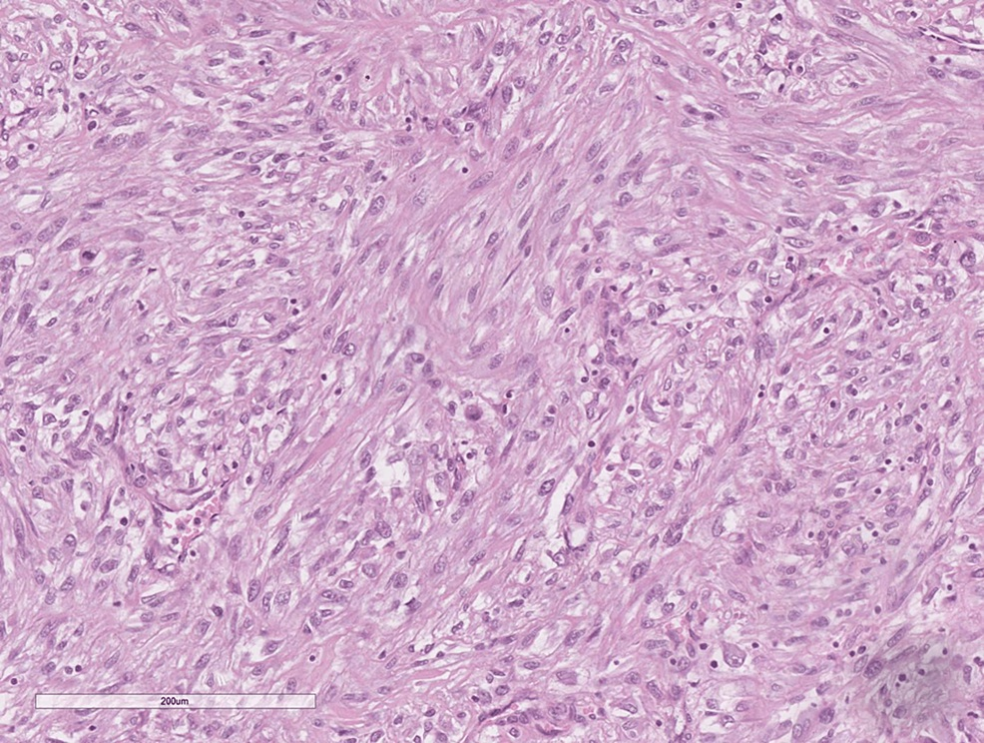
Histopathology of the tumor, H&E stain, original magnification 200x H&E: hematoxylin and eosin

**Figure 6:**
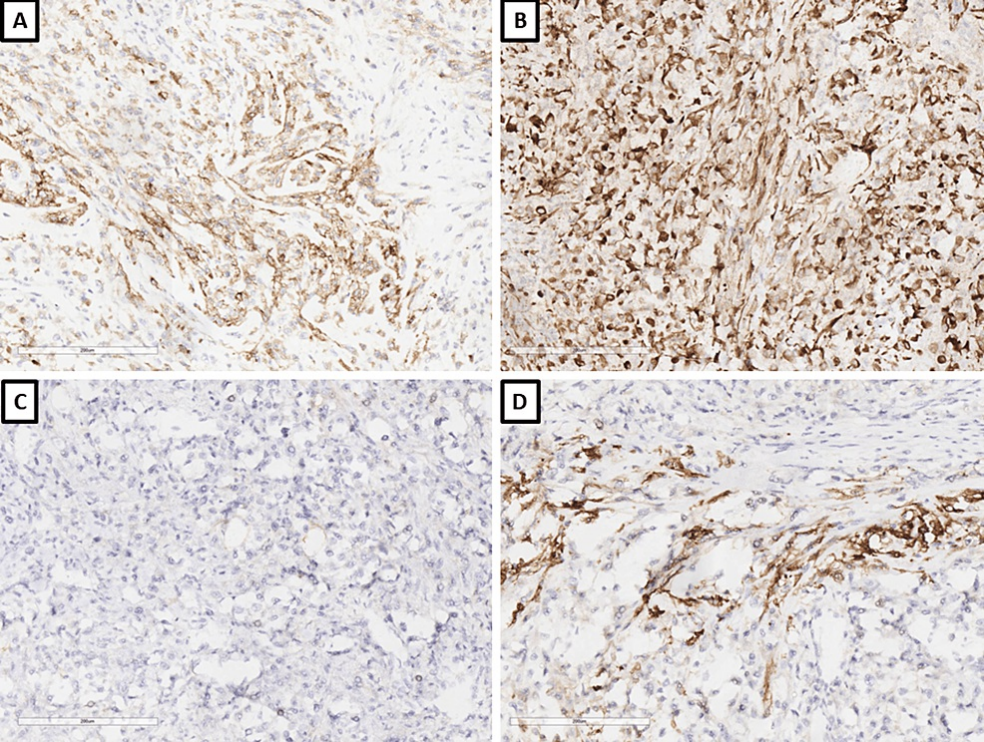
Immunophenotype of the tumor, original magnifications x200 A: CD10; B: CK AE1/AE3; C: CK7; D: EMA CD: cluster of differentiation; CK AE1/AE3: pan-cytokeratin; CK7: cytokeratin 7; EMA: epithelial membrane antigen

**Figure 7:**
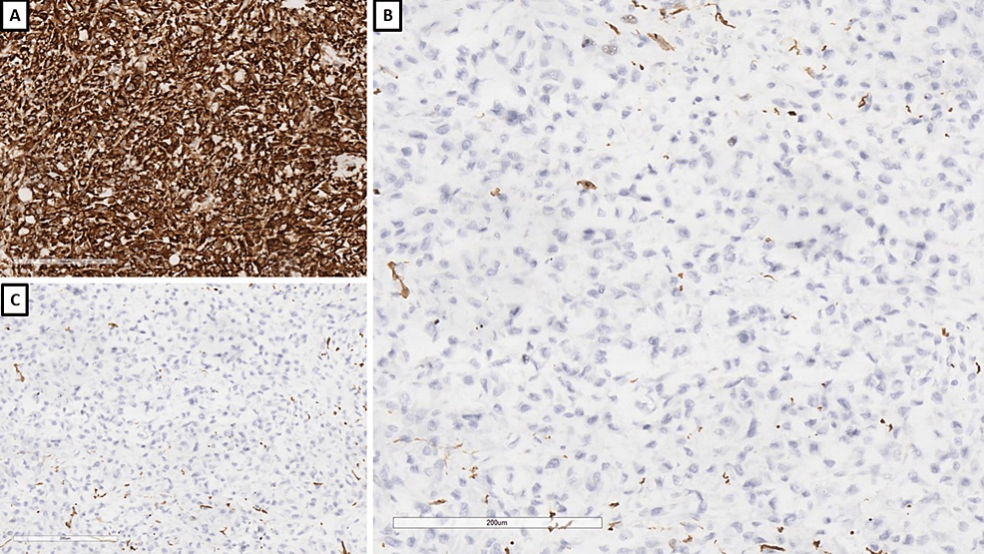
Immunophenotype of the tumor, original magnifications x200 A: caldesmon; B: SMA; C: desmin SMA: smooth muscle actin

Histologically, the tumor grew as intertwining bundles of spindle cells with polymorphic hyperchromic nuclei and prominent nucleoli and had extensive areas with necrosis (Figure 5). Immunohistochemically, the tumor diffusely expressed CK AE1/AE3 and caldesmon and had a patchy reaction for EMA and CD10 (Figures 6-7). The SMA, desmin, CD117, and CK7 reactions were negative (Figures 6-7). The tumor was interpreted as a spindle cell variant of clear cell RCC and graded as per the International Society of Urological Pathology (ISUP) as ISUP G4, and staging was referred back to the institution in which the patient underwent surgery due to insufficient data on the size and spread. The staging was performed by the oncologic committee of the initial institution (due to the lack of relevant data), with pathological staging performed by us suggesting at least a pT3a due to evidence invasion into the renal hilum.

## Discussion

Primary leiomyosarcoma is a rare kidney tumor, accounting for 1-2% of all kidney tumors. Histologically, primary leiomyosarcoma of the kidney must be differentiated from sarcomatoid RCC, sarcomatoid urothelial carcinoma of the renal pelvis or calyces, leiomyoma, angiomyosarcoma, and angiomyolipoma. Distinguishing leiomyosarcoma from leiomyoma is not difficult because mitoses and extensive necrosis are present only in malignant tumors, although cellular pleomorphism can be seen in both. In renal angiomyolipoma, bundles of smooth muscle cells are mixed with mature fat and thick-walled blood vessels.

The high frequency of expression of epithelial markers in leiomyosarcoma and their expression in other stem cell neoplasms leads to serious differential diagnostic problems. Careful consideration of cellular morphology and interpretation of immunohistochemical markers is required to make the correct diagnosis [12].

We offer the following differential diagnostic immunohistochemical markers to differentiate these tumors (Table 1).

**Table 1:** Immunophenotypic differential diagnosis of renal spindle cell tumors Adapted from Ref. [2]. CK AE1/AE3: pan-cytokeratin; EMA: epithelial membrane antigen; SMA: smooth muscle actin; HMB-45: human melanoma black 45; S100: soluble 100 protein; ER: estrogen receptor; PR: progesterone receptor; CD: cluster of differentiation; CK7: cytokeratin 7; GATA3: guanine-adenine-thymine-adenine (GATA) nucleotide sequence-binding protein 3; RCC: renal cell carcinoma; PAX8: paired box gene 8

Marker	Leiomyosarcoma	Sarcomatoid variant of urothelial carcinoma	Sarcomatoid variant of renal cell carcinoma	Leiomyoma	Angiosarcoma	Angiomyolipoma
Mitotic index	High	High	High	Low	High	Low
CK AE1/AE3	Positive in around 40% of cases	Positive in approximately 70% of cases	Positive	Negative	Positive in epithelioid angiosarcoma	Negative
EMA	Positive in approximately 40% of cases	Positive	Positive in around 50% of cases	Negative	Positive in epithelioid angiosarcoma	Negative
Vimentin	Positive	Positive	Positive in approximately 60% of cases	Positive	Positive	Positive
SMA	Usually positive	Positive in approximately 70% of cases	Negative	Positive	Negative	Positive in vessels
h-Caldesmon	Positive	Negative	Negative	Positive	Negative	Negative
Desmin	Positive	Negative	Negative	Positive	Negative	May be positive
HMB-45	Negative	Negative	Negative	Negative	Negative	Varying rates of positivity in the different components
S100	Negative	Usually positive	Usually positive	Negative	Negative	Positive in adipocytic component
ER	Negative	Negative	Negative	Positive	Negative	Positive
PR	Negative	Negative	Negative	Positive	Negative	Positive
CD68	Negative	Negative	Negative	Negative	Negative	May be positive
CK7	Negative	Positive	Positive	Negative	Negative	Negative
GATA3	Negative	Positive	Negative	Negative	Negative	Negative
RCC	Negative	Positive	Positive	Negative	Negative	Negative
PAX8	Negative	Highly variable	Positive	Negative	Negative	Negative
CD10	Negative	Negative	Positive	Negative	Negative	Negative
CD117	Negative	Negative	Negative	Negative	Negative	Varying rates of positivity in the different components
Melan A	Negative	Negative	Negative	Negative	Negative	Varying rates of positivity in the different components
CD34	Negative	Negative	Negative	Negative	Positive	Positive
Uroplakin II	Negative	Positive	Negative	Negative	Negative	Negative

As seen in Table 1, the differential diagnosis of spindle cell neoplasms within the kidney which, although rare, is extremely broad, and few definitive markers are available. In these instances, the usual differential approach with mesenchymal and epithelial markers is of little use due to the high chance of mesenchymal tumors being positive for epithelial markers, epithelial markers being negative in epithelial tumors, and vice versa. Twp of the few markers with a high degree of confidence in these instances are RCC and guanine-adenine-thymine-adenine nucleotide sequence-binding protein 3 (GATA3), which are highly specific for renal cell and urothelial malignancies, respectively; however, their rate of confidence is not absolute, especially in the case of GATA3 as it is also positive in a myriad of other malignant conditions, predominantly epithelial in origin [13]. Desmin and caldesmon carry relatively the same specificity for mesenchymal tumors but are not definitive for the type of malignancy. Based on the immunoprofile of the tumor, it is possible to pinpoint the groups precisely, still rarely the nosological unit itself, with the final diagnosis performed with a combination of positive and negative markers on immunohistochemistry and morphological hallmarks and features.

In the case of differentiating spindle cell type of clear cell RCC, the WHO suggests that, even in focal renal cell marker positivity or even focal epithelial marker positivity on immunohistochemistry, the tumor is to be accepted as epithelial in the absence of positive mesenchymal markers [1]. Even still, the diagnosis remains difficult and is essentially a combination of interpreting the histopathology with the variable combination of expression patterns of semi-specific antibodies on immunohistochemistry [2,11].

As these are malignant conditions, the final histopathological diagnosis is vital as treatment (chemo and radiotherapy) is tumor-specific, and different groups of tumors have different progression patterns and prognosis, as highlighted by the fast and significant local recurrence of the first tumor.

## Conclusions

As highlighted by the two cases presented, spindle cell neoplasms of the kidney are nosological units challenging to diagnose due to their rare incidence, non-specific histopathology, lack of specific immunohistochemical markers, and broad differential diagnosis with one another. Furthermore, the consequences of misdiagnosis are severe as the progression, metastasis, and disease-related death rates vary significantly between the separate groups - one of the depicted patients is stable and non-progressing. At the same time, the other one has developed local recurrence and distant metastasis for a relatively short period of time.
